# MORAL ENHANCEMENT AND FREEDOM

**DOI:** 10.1111/j.1467-8519.2010.01854.x

**Published:** 2010-12-07

**Authors:** John Harris

**Keywords:** moral enhancement, cognitive enhancement, freedom, God

## Abstract

This paper identifies human enhancement as one of the most significant areas of bioethical interest in the last twenty years. It discusses in more detail one area, namely moral enhancement, which is generating significant contemporary interest. The author argues that so far from being susceptible to new forms of high tech manipulation, either genetic, chemical, surgical or neurological, the only reliable methods of moral enhancement, either now or for the foreseeable future, are either those that have been in human and animal use for millennia, namely socialization, education and parental supervision or those high tech methods that are general in their application. By that is meant those forms of cognitive enhancement that operate across a wide range of cognitive abilities and do not target specifically ‘ethical’ capacities. The paper analyses the work of some of the leading contemporary advocates of moral enhancement and finds that in so far as they identify moral qualities or moral emotions for enhancement they have little prospect of success.

## I. THE ARGUMENT

God had important things to say on the subject of moral enhancement. If God's feelings on the subject have been reliably reported by John Milton, the verbatim account to be found in *Paradise Lost*^1^ is important because it contains, in a most concise form, many of the most cogent reasons for suspicion as to the viability of moral enhancement as a coherent project, at least as it is being understood in the emerging literature.

Another good reason to listen to^2^ Milton's God is his insistence on the obligation that we all have to take responsibility for ourselves and for our world and in contradiction of so many recent writers who claim the hubris of attempting to do so exposes us to a myriad of dangers.^3^ Equally the failure to exercise such responsibility is far from the path of safety^4^ and hot baths.^5^

The recent history of bioethics is marked by its commitment, by a move from ethics as etiquette to ethics as engagement.^6^ This is all the more vital because from many sources we are receiving warnings of the necessity for early and decisive action to save both humanity and indeed the planet. Climate change, new diseases such as Avian and Swine Flu, the various forms of Creutzfeldt-Jacob Disease (CJD) and HIV/AIDS, the population explosion, and increasingly diffused access to weapons of mass destruction all urgently require solutions and are all posing extraordinarily difficult problems and presenting unprecedented dangers. While bioethics is hardly plausible as the saviour science for all these ills, it does have,and has exercised, a vital role in both highlighting problems and in clearing away many of the tenacious and bad arguments that are constantly produced for avoiding or postponing radical solutions.

Against this background, Moral Enhancement is coming to the forefront of bioethical scholarship for an interesting combination of reasons. On the one hand it harnesses one of the central areas of traditional philosophy, namely ethics, to very recent developments in neuroscience and in psychology and on another it highlights the centrality of both human curiosity and our passion for self improvement to our decisions about, and hopes for, the future of humanity. Thus moral enhancement combines cutting-edge science with mainstream philosophy and with the hopes and fears of ordinary people. In what follows I hope to show why the idea of moral enhancement is being fundamentally misunderstood by many of those interested in further research in this field, and in particular, why mistakes about the nature of both the opportunities it offers and the very nature of ‘right conduct’ are presenting dangers for the present and the future of humanity.

But let's return (or turn) to God for a moment. Famously, in Book III of *Paradise Lost* Milton reports God saying to his ‘Only begotten Son’ that if man is perverted by the ‘false guile’ of Satan he has only himself to blame:

… … … … … whose fault?

Whose but his own? Ingrate, he had of me

All he could have; I made him just and right,

Sufficient to have stood, though free to fall.^7^

These lines have inspired many writers about the human condition and about the precious nature of freedom and in particular free will. William Golding echoed these famous lines and discussed their theme in his novel *Free Fall.*^8^ I first read *Free Fall* as an undergraduate in the 1960s^9^ (and it was Golding that pointed me to Milton). Golding asks two crucial questions in that book: ‘when did I lose my freedom?’, and ‘how did I lose my freedom?’ Here is how they are posed on the first page of *Free Fall*:

When did I lose my freedom? For once I was free. I had power to choose. The mechanics of cause and effect is statistical probability yet surely sometimes we operate below or beyond that threshold. Free will cannot be debated but only experienced, like a colour or the taste of potatoes. I remember one such experience. I was very small and I was sitting on the stone surround of the pool and fountain in the centre of the park. There was bright sunlight, banks of red and blue flowers, green lawn. There was no guilt but only the plash and splatter of the fountain at the centre. … The gravelled paths of the park radiated from me: all at once I was overcome by a new knowledge. I could take whichever I would of these paths. There was nothing to draw me down one more than the other. I danced down one for joy in the taste of potatoes. I was free I had chosen.^10^

Leaving aside Golding's rather suspect views about statistical probability and the assertion that free will cannot be debated (which is demonstrably contradicted in the passage just quoted), Golding vividly illustrates a feeling that surely everyone has had, the feeling of what it is like to be free in an existential sense.^11^ With the exhilaration of that feeling, I hope, coursing through our veins (and not, I hope, clouding our judgement) let's return to Milton.

When God says of man that ‘he had of me all he could have’ he qualifies this in two ways. Firstly by the vainglorious claim ‘I made him just and right’, and second by a wonderful analysis of freedom: ‘sufficient to have stood, though free to fall’. Milton's God was certainly overestimating her role in making humankind just, right and all the rest, but nature, or more particularly, evolution, has done most of this for us. We have certainly evolved to have a vigorous sense of justice and right, that is, with a virtuous sense of morality. God was, of course, speaking of the fall from Grace when congratulating herself on making man ‘sufficient to have stood though free to fall’; she was underlining the sort of existential freedom Golding spoke of which allows us the exhilaration and joy of choosing (and changing at will) our own path through life. And while we are free to allow others to do this for us and to be tempted and to fall, or be bullied, persuaded or cajoled into falling, we have the wherewithal to stand if we choose. So that when Milton has God say mankind ‘had of me all he could have’, he is pointing out that while his God could have made falling impossible for us, even God could not have done so and left us free. Autonomy surely requires not only the possibility of falling but the freedom to choose to fall, and that same autonomy gives us self-sufficiency; ‘sufficient to have stood though free to fall.’

It would be tempting to conclude at this point that we humans, although we need many forms of enhancement and often desire much more enhancement than we need, do not need and are irrational to seek, specifically *moral* enhancement. This is because we already have not only an extensive moral endowment but because the ways being canvassed to enhance that endowment are unlikely to leave us sufficient to stand though free to fall. However that would not be quite right either. There are many very attractive and effective forms of moral development including enhancement, available; it is simply that they are not the ones so far being spoken of^12^ as either relevant to moral or to neuro-enhancement.

These tried and tested methods include the traditional ones of bringing children up to know the difference between right and wrong, to avoid inflicting pain or suffering on or doing harm to others; and instilling in them habits of respect for others. These modes of respect include altruism, sensitivity and consideration and perhaps above all of being able to put ourselves in others' shoes so that we not only understand, but imaginatively experience, what it might be like to be on the receiving end of the conduct of others. Equally, more general education, including self education, wide reading and engagement with the world and with ways in which the world is mediated, (including mass media, computers and the internet), are powerful tools of moral development and improvement or enhancement. These must include, of course, sophisticated understanding of cause and effect, in particular of the ways in which to allow things to occur is as effective a way of determining the state of the world as is making positive interventions.^13^

### Moral enhancement^14^

In considering moral enhancement, the first questions to ask are: what is moral enhancement and what does it have to do with ethical knowledge, if there is such a thing, or with ethical expertise; and what do all of these have to do with knowledge of ethics or morality?

One thing we can say with confidence is that ethical expertise is not ‘being better at being good’, rather it is being better at knowing the good and understanding what is likely to conduce to the good. The space between knowing the good and doing the good is a region entirely inhabited by freedom. Knowledge of the good is sufficiency to have stood, but freedom to fall is all. Without the freedom to fall, good cannot be a choice; and freedom disappears and along with it virtue. There is no virtue in doing what you must.

Those with the insight, sympathy, empathy understanding and knowledge to have formed clear ideas of what might conduce to the good are not necessarily better at doing good in any of the ways in which this is possible, including of course making the world a better place. There are many reasons for this and we have space only for a few.

Some of these are to do with a problem understood since at least classical Greece: the problem of ‘akrasia’ or weakness of will, one form of which was brilliantly summarized by George Bernard Shaw, when he defined virtue as ‘insufficient temptation’. We know how lamentably bad we are at doing what we know we should. But equally problematic is the fact that this is not, or not wholly, due to lack of moral fibre or resolution or firmness of purpose. Rather, and again only very partially, it is because we have many purposes, many things to do and experience and many priorities, of which being good we hope is a part because we hope we do all of these things in a good, a moral way. But of course because we are doing these other things, hopefully with benevolence and good will and good intentions, it is the other purposes for which we also do them that are often, if not more important, at least more at the centre of our attention.

A very fundamental problem, which has not been much discussed in the literature on moral enhancement, is that the sorts of traits or dispositions that seem to lead to wickedness or immorality are also the very same ones required not only for virtue but for any sort of moral life at all.

Tom Douglas, who has a good claim to be one of the ‘grandfathers’ of moral enhancement, has defined the most promising form of this field as ‘an enhancement that will expectably leave the enhanced person with morally better motives than she had previously’.^15^ Noting substantial difficulties in identifying ‘good motives’ let alone thinking about how, other than through early education and imaginative engagement with others, or thinking about how to manipulate or improve them, Douglas adopts the interesting expedient of trying to identify what he calls ‘counter-moral emotions'. He identifies at least two: ‘a strong aversion to certain racial groups' and ‘the impulse towards violent aggression’. Douglas commits himself to the belief that ‘there are some emotions such that a reduction in the degree to which an agent experiences those emotions would, under some circumstances, constitute a moral enhancement’.^16^

There are two substantial problems with this, albeit highly creative, approach. The first is that it seems unlikely that, for example, an aversion to certain racial groups, or to one or more gender or sexual orientation is simply a ‘brute’ reaction, a sort of visceral response, as perhaps isan aversion to spiders. Rather it is likely to be based on false beliefs about those racial or sexual groups and or an inability to see why it might be a problem to generalize recklessly from particular cases. In short prejudice, as well as rationality, usually has cognitive content and often makes factual claims. Beliefs with cognitive content are for example beliefs that X is true or Y is false, that A is a danger and B is not, that C is good and D is evil, they are explained by the people that have them in terms of beliefs and ideas, including beliefs about facts which may be, and therefore can often be shown to be, true or false.

The most obvious countermeasure to false beliefs and prejudices is a combination of rationality and education, possibly assisted by various other forms of cognitive enhancement, in addition to courses or sources of education and logic.

Reasons have been advanced for denying or at least circumventing the cognitive elements of certain ‘immoral beliefs’. Ingmar Perrson and Julian Savulescu^17^ refer to research which reports that ‘people encode the race of each individual they encounter, and do so via computational processes that appear to be both automatic and mandatory. Encoding by race is a by-product of cognitive machinery that evolved to detect coalitional alliances.’^18^ While this may be true and while this encoding might be susceptible to disruption, there are some problems with seeing this as a key to moral enhancement. Racism still remains widespread but is almost everywhere deplored and in many countries is also against the law. And of course it is racist behaviour, not racist beliefs that are the problem, or the main problem.^19^ The most important thing about the prejudices that most, perhaps all of us, have in one form or another, is to recognize them and learn to be ashamed of them and above all not to act on them. The neutralization of the worst effects of racist beliefs is thus probably improved by cognitive enhancement. Moreover, it seems likely that racism affects, in a virulent form, only a minority of the world's population; and yet all of us have the encoding, so one might think that the encoding cannot be that powerful. Racism has been further reduced dramatically in the last hundred years by forms of moral enhancement including education, public disapproval, knowledge acquisition and legislation. We thus have a very effective blueprint for the sorts of ways in which we can reduce and hopefully eventually effectively eradicate racism. The blueprint provides a good measure of the effectiveness of these means and good reason to believe that racism can be defeated by such means without resorting to biological or genetic measures which might have unwanted effects. In the present case such an unwanted effect might be to weaken kinship ties or other ties unconnected with race, for reasons similar to those we are about to discuss.

Moreover, Perrson and Savulescu suggest that having:

suggested that the core moral dispositions … have a biological basis and, thus, in principle should be within the reach of biomedical and genetic treatment, the next question is to what extent such treatment is possible in practice. To this question the answer seems to be: only to a very small extent.^20^

To return to Douglas, the second problem with his account is that we would need to be pretty sure that ‘the reduction in the degree to which the emotion was experienced’ could be precisely targeted only on strong aversions to things it is bad to have strong aversions to and not on things to which strong aversions are constitutive of sound morality. This problem was effectively articulated by Peter Strawson in a famous essay entitled ‘Freedom and Resentment’.^21^ Strawson was not of course concerned with moral enhancement, but rather with the problem of free will. And in the course of combating some absurd forms of determinism, he points out that certain strong emotions, including aversions, are an essential and even desirable part of valuable emotions, motives or attitudes to others. Could we in short have the sorts of feelings that are appropriate and indeed, it might be argued, necessary to morality, if we did not feel a strong aversion to, for example, someone who deliberately and unjustifiably killed or tortured those we love?

While Douglas is right when he claims ‘there are some emotions such that a reduction in the degree to which an agent experiences those emotions would, under some circumstances, constitute a moral enhancement’^22^ this is a very modest claim indeed, and I for one am sceptical that we would ever have available an intervention capable of targeting aversions to the wicked rather than the good. Of course if ever we do have the prospect of such precise and unequivocally good producing interventions, I will welcome them. But I remain doubtful and remain worried about the prospect of weakening possibly essential and essentially moral responses. This is a ‘baby and bathwater’ problem which may prove soluble; I hope it will, but fear it may be intractable.

As we have seen, there are substantial issues of liberty which would also need to be resolved and which could conceivably be threatened by any measures that make the freedom to do immoral things impossible, rather than simply making the doing of them wrong and giving us moral, legal and prudential reasons to refrain.

## II. THE ANALYSIS

### Purity and danger

Ingmar Perrson and Julian Savulescu have produced a characteristically bold and simultaneously intriguing and a worrying manifesto for the urgency and importance of moral enhancement. In a paper whose title sums up the agenda,^23^ they are pessimistic, to the point of paranoia,^24^ about the merits of cognitive enhancement and seem to argue that efforts to improve cognitive powers and capacities should be put on hold until moral enhancement is perfected, infallible, and made not only universally available but comprehensively mandatory. One problem with such an approach is that there are good reasons (which we have reviewed and will return to in due course) to believe moral enhancement must, in large part consist of cognitive enhancement.

Persson and Savulescu summarize their argument in five main claims:

1. It is comparatively easy to cause great harm, much easier than to benefit to the same extent.
2. With the progress of science, which would be speeded up by cognitive enhancement it becomes increasingly possible for small groups of people, or even single individuals, to cause great harms to millions of people, e.g. by means of nuclear or biological weapons of mass destruction.
3. Even if only a tiny fraction of humanity is immoral enough to want to cause large scale harm by weapons of mass destruction in their possession, there are bound to be some such people in a huge human population, as on Earth, unless humanity is extensively morally enhanced. (Or the human population is drastically reduced, or there is mass genetic screening and selection, though we take it that there is no morally acceptable way of achieving these sufficiently effectively.)
4. A moral enhancement of the magnitude required to ensure that this will not happen is not sufficiently possible at present and is not likely to be possible in the near future.
5. Therefore, the progress of science is in one respect for the worse by making likelier the misuse of ever more effective weapons of mass destruction, and this badness is increased if scientific progress is speeded up by cognitive enhancement, until effective means of moral enhancement are found and applied.

Perrson and Savulescu conclude that:

If safe moral enhancements are ever developed, there are strong reasons to believe that their use should be obligatory … That is, safe, effective moral enhancement would be compulsory.

Now of course the mischief is in the meaning … in this case of the words ‘safe and effective’ but before seeing just how much mischief might be possible it is worth taking a moment to examine the plausibility of Perrson and Savulescu's five main claims.

### First Claim

1. It is comparatively easy to cause great harm, much easier than to benefit to the same extent

This seems superficially true, so that when Perrson and Savulescu draw attention to the Virginia Tech killings in 2007 and say:

Seung-Hui Cho killed 32 people in the worst civil shooting in US history. Cho used two semiautomatic handguns. The actual killings took place in a couple of minutes. It is almost never possible to save 32 lives in the same period of time.^25^

we are inclined to accept it at face value.

But when we stop nodding at the obvious, but limited, relevance of such an example and examine it, this claim shows itself to be totally implausible. Raising the alarm when a fire is noticed in a school so that the building can be successfully evacuated or overpowering hijackers or terrorists who would destroy a plane in flight, often saves as many and usually more lives in as many minutes. So that when Person and Savulescu say: ‘It is almost never possible to save 32 lives in the same period of time’ I am sorry to have to say that this is manifestly absurd.

On Saturday 26 December 2009 Umar Abdul Mutallab tried to set off a bomb on flight 253 carrying 290 people while it was attempting to land in Detroit. Jasper Schuringa became an international hero for thwarting this attempt and probably saving every life on the plane. This is the report of the incident from *The Observer,* a London newspaper, of the following day:

When [it] went off, everybody panicked,’ said Jasper Schuringa, a Dutch film director travelling to the US to visit friends. ‘Then someone screamed, ‘Fire! Fire!’ I saw smoke rising from a seat … I didn't hesitate. I just jumped.’ Schuringa said he heard a sound similar to a firework going off and looked across the aisle at the suspect who had a blanket on his lap attempting to ignite an object he was holding. ‘It was smoking and there were flames coming from beneath his legs,’ he said. ‘I searched on his body parts and he had his pants open. He had something strapped to his legs.’

Schuringa and the cabin crew then dragged Mutallab, a 23-year-old Nigerian, to the front of the plane, where he was restrained until landing. Mutallab reportedly told intelligence agents who began interrogating him after he was taken to hospital strapped to a stretcher that he had an explosive powder strapped to his leg. He was trying to set off the device with a syringe filled with liquid.^26^

We are obviously not going to quibble over the meaning of the words ‘same period of time’. I am sure Mr Schuringa did not time the event, but the issue is the plausibility of the claim ‘It is comparatively easy to cause great harm, much easier than to benefit to the same extent.’ The example of Flight 253^27^ shows that 9 times the number of people, as in the Perrson and Savulescu example, can be saved in a comparable period of time; and this is not an isolated example. The case of the infamous shoe bomber who was overpowered by crew and passengers while trying to set off a bomb and which anyone who has had to remove their shoes at airport security will remember with affection,^28^ was similar, and there are many more.

More obviously, the voting of huge sums of money for famine relief or aid work following a tsunami or earthquake, or to allocate vaccines (for example the provision of 35 million doses of the Tamiflu vaccine for the UK population against a pandemic)^29^ is the work of a few minutes, but can save thousands, even millions of lives. In the event, of course, the influenza pandemic was much milder than feared and most of the anti-viral reserves remain for another day. Vaccine programmes, while not instantly implementable, are quick and dramatic ways to save millions of lives. Whatever the death toll of a disaster, once methods of preventing a recurrence are found, the implementation of those methods almost certainly saves as many if not more lives as might have been threatened by the disaster. This is because preventive measures probably forestall not only this year's disaster but next years' and the following years as well. For if the preventive measures are permanent, as is likely in the case of smallpox and polio for example, then arguably an indefinite time sensitive multiplication of the death toll relates plausibly and proportionately to the lives saved. Whether or not this is an acceptable basis for a precise calculation, it is surely unlikely that the lives saved will be less than those previously lost in a permanently prevented pandemic. Thus:

Smallpox continued to ravage Europe, Asia, and Africa for centuries. In Europe, near the end of the eighteenth century, the disease accounted for nearly 400,000 deaths each year, including five kings. Of those surviving, one-third were blinded. The worldwide death toll was staggering and continued well into the twentieth century, where mortality has been estimated at 300 to 500 million. This number vastly exceeds the combined total of deaths in all world wars.^30^

And arguably the initiation of the WHO plan to eradicate smallpox saved at least as great a proportion of the world's population as that estimated to be threatened by it.

In 1967, when WHO launched an intensified plan to eradicate smallpox, the ‘ancient scourge’ threatened 60% of the world's population, killed every fourth victim, scarred or blinded most survivors, and eluded any form of treatment.^31^

Since the last century, as Perrson and Savulescu confirm, smallpox is regarded as entirely eradicated. So much for the claim that: ‘It is comparatively easy to cause great harm, much easier than to benefit to the same extent’! Perrson and Savulescu can certainly be forgiven for this error, it is all too easy to accept a dramatic cliché which seems to illustrate something one is tempted to believe; and I have done so many times myself. It is however useful to lay this particular ‘canard’ to rest once and for all.

Perrson and Savulescu quite rightly make great play with the dangers of biological weapons and the possibilities of bioterrorism.

The polio virus has now been artificially constructed … More frighteningly scientists have modified mousepox to make it lethal in 100% of mice … Voltaire estimated that smallpox killed around 20% of the French population in his day. It was eradicated last century from the globe by vaccination … Genetic engineering of smallpox could create a new strain which would wipe out all or most of humanity.

So it could, and cognitively enhanced science might create a vaccine in time to prevent it. This seems a telling example; but what does it tell? The answer is that it tells rather against the Perrson/Savulescu thesis than for it. First it shows that just as a disease like smallpox is an effective killer, vaccines against it are equally successful ways of saving lives. How long did it take to kill 20% of the French population in Voltaire's day? How long did it take to save those same numbers with a vaccine? These are complex questions and because of ambiguities about when a lethal agent operates and when a protective measure actually does the protecting, they are probably not even answerable questions.

But if we compare the time taken by Seung-Hui Cho to kill 32 people and the time it would take to administer, say, 32 doses of polio vaccine via sugar lump to a queue of children, the time difference would not be significant. Add to this the numbers killed by polio and the numbers vaccinated, and we can see that it is often comparatively easy and not time-consuming to save great numbers of lives.

Of course it might be claimed that the development of effective vaccines, for example against polio and smallpox, took a great deal of time; but so did the development of semiautomatic handguns. From the first use of firearms, somewhere between China in the ninth century and medieval Europe in the thirteenth century, to today is a very long time indeed. The fact that, once developed, both vaccines and semiautomatic firearms are fast acting, emphasizes the similarities rather than the differences.

What seems clear is that the time the killing or the saving takes is trivial compared with the effectiveness of each. No generalizations to the effect that damage is always or even often quicker than repair, or prevention slower in taking effect than what it prevents, are helpful in addressing the potency of dangers or the probability of defences against them being effective. We can be confident in our conclusion that claim one is false; not least because it depends on upholding a version of the acts/omissions distinction which I am sure that Savulescu, at least, rejects. For if a mad or bad individual can destroy the world instantly by setting off a doomsday machine, then a good consequentialist can save the world as quickly by killing him (or morally enhancing him) the moment before he can do so or at any time before that! And this sort of prevention is a recurrent theme of Savulescu's work.

### Claims 2 and 3 need to be considered together

2. With the progress of science, which would be speeded up by cognitive enhancement it becomes increasingly possible for small groups of people, or even single individuals, to cause great harms to millions of people, e.g. by means of nuclear or biological weapons of mass destruction.
3. Even if only a tiny fraction of humanity is immoral enough to want to cause large scale harm by weapons of mass destruction in their possession, there are bound to be some such people in a huge human population, as on Earth, unless humanity is extensively morally enhanced …

Essentially this is the claim that it only takes one bad man to spoil things for the rest of us. This may well be true, in the sense that its possibility cannot be ruled out, but it is not just the wicked that present problems of this sort. Moreover, it is not clear either that speeding up the progress of science through cognitive enhancement exacerbates the process, nor that moral enhancement has much prospect of eradicating this possibility; indeed the reverse may be true. We need to remember that any tool or technology can be abused or misused and that accidents and negligence can already routinely cause or threaten,; harms on a massive scale (Three Mile Island, Chernobyl).

If, and in so far as, it is true that scientific progress increases the power of individuals to do harm, it is not clear that moral enhancement, if and when it might be possible to imagine it to have become ‘safe’ and ‘effective’, will much reduce this danger. Although I am sure that Perrson and Savulescu would acknowledge that mad, as well as bad, individuals can cause harm, they talk throughout their paper almost exclusively as if the danger was from wickedness, perhaps because they want to encourage the prioritization of moral enhancement as a field of study.

### The village idiot

Now add to this that the danger comes not simply from the malevolent, but from another important category of disastrous individuals. Perrson and Savulescu refer to the work of Martin Rees. Rees is famous for (among many other things) his elevation of the ‘village idiot’ to global status. In his fascinating book *Our Final Century*^32^ Rees catalogues the disasters which might, if the causes of them are not addressed, lead to the end of human life on this planet. Rees considers the role of malevolence or wickedness but also warns of the dangers posed by incompetence or stupidity.

We are entering an era when a single person can, by one clandestine act, cause millions of deaths or render a city uninhabitable for years, and when a malfunction in cyberspace can cause havoc worldwide to a significant segment of the economy: air transport, power generation, or the financial system. Indeed disaster could be caused by someone who is merely incompetent rather than malign.^33^

Rees glossed these remarks at the Hay festival in 2006: ‘in a global village there will be global village idiots, just one could be too many,’ and he concluded ‘I think there is a real concern about whether our civilization can be safe-guarded without us having to sacrifice too much in terms of privacy, diversity and individualism’.^34^ I agree with Rees about this, and would add dangers to liberty and autonomy. But I run too far ahead; let's complete our discussion of Perrson and Savulescu.

### Claims 4 and 5 need to be considered together

4. A moral enhancement of the magnitude required to ensure that this will not happen is not sufficiently possible at present and is not likely to be possible in the near future.
5. Therefore, the progress of science is in one respect for the worse by making likelier the misuse of ever more effective weapons of mass destruction, and this badness is increased if scientific progress is speeded up by cognitive enhancement, until effective means of moral enhancement are found and applied.

These final two claims are interesting. The first that moral enhancement by new and more radical means than education, knowledge acquisition and scientific progress is at best a long way off seems right to me; but the alleged sequitur is definitely of the ‘non’ variety. One of the reasons for this is that claim 4 not only notes how far off moral enhancement of any new kind is likely to be, but refers to a ‘moral enhancement of the magnitude required to ensure that’ disaster is avoided. There are many reasons to believe that such ‘ensurance’ and the assurance that follows from it, are impossible, not least because failures in any human intervention are not only possible but are arguably inevitable. There is no such thing as *ensuring* safety.

Even more significant are the costs of delay. While we wait patiently with Perrson and Savulescu for the mid to far future perfection of genetic or biological moral enhancement, and while we put on hold the cognitive enhancement that might accelerate scientific advance and the discovery and innovation it produces, ‘stuff’ or even events happen!^35^ That stuff will be the minute-by-minute, day-by-day accumulation of premature death and suffering from causes that cognitive enhancement and the resulting innovation might have prevented.

In my book *Violence and Responsibility*,^36^ published thirty years ago now, I was concerned with our responsibility for harms which we might prevent or might have prevented. I insisted that, for example, the violence of political change must be evaluated against what the social historian Barrington Moore Jr called ‘the violence of normal times’. ‘To dwell on the horrors of revolutionary violence while forgetting that of “normal” times’ he said is ‘merely partisan hypocrisy’.^37^ Barrington Moore Jr warned that the death toll of, for example the revolutionary terror of the French Revolution must be seen as a response to ‘the prevailing social order’ which ‘always grinds out its toll of unnecessary death year after year’.^38^ We are attuned now to be sensitive to the costs of delay in instituting not only social reform that might prevent unnecessary death and suffering, but the delays that result from failures to turn discovery rapidly into innovation, and to ensure that innovation results in products in the clinic and the marketplace that will save and ameliorate lives. Perrson and Savulescu refer to the work of Jonathan Glover, although not in this regard, but they might also have referred to earlier work of Julian Savulescu. It is an irony that Savulescu is here advocating the sort of delays in adopting new technology and medical innovation which certainly will cost lives, delays of the sort that in the past he has been vigilant to oppose.^39^ Of course if haste will cost more lives than delay we have good reasons for a precautionary approach. In the present case, as Perrson and Savulescu admit, there is not only no immediate prospect of moral enhancement but we have literally no idea how long (if ever) it will take to perfect. On the other hand we have daily evidence of the record of science and technology in preventing and treating disease and premature death and in dramatic increases in life expectancy.

Moreover if Martin Rees is right this cannot be *ensured* because it is unlikely that moral enhancement will affect the proverbial ‘village idiot’, nor the sort of disaster that might result from negligence or miscalculation. Rees's book cites many examples of how much room there is for the disastrous miscalculation of the level of risk inherent in many apparently benign or ‘morally neutral’ technologies. The question then becomes one of whether forgoing the benefits that might accrue from accelerating science via cognitive enhancement, including the rapid development of antidotes to engineered diseases and other bio-weapons and biohazards, better insights into how to combat the worst effects of climate change, and reliable methods of predicting asteroid strikes and developing methods of diverting the asteroids, to identify just a few of the dangers that we may hope will prove amenable to a scientific or technological ‘fix’. While we can be sure of none of these things, it would not simply be a brave man but surely a reckless one, who would bet against the overall utility of scientific advance and cognitive enhancement.

Julian Savulescu is one of the smartest people I know. Until this moment I had no reason to think of him as, for this reason, also one of the most dangerous. Should I also begin to look on my most talented students with equal suspicion and do my best to sabotage their cognitive advancement? I hope I have found reasons in this paper both to continue to admire Julian, and continue to encourage my best students. Indeed as Elizabeth Fenton has pointed out ‘it is difficult not to take [Perrson's and Savulescu's pessimism] to imply that unless and until we further understand moral enhancement, we should try to slow scientific progress’.^40^ She might have gone further and pointed out that their extreme risk aversion would justify not only retarding scientific progress but retarding the cognitive powers of people as well.

### Milton revisited

Although Perrson and Savulescu say:

True, there are also respects in which scientific progress accelerated by cognitive enhancement would be for the better, by protecting us against threats posed by asteroids, epidemics etc. We have not attempted to settle definitely the balance between these good and bad respects.

this is disingenuous and is at odds with what they say elsewhere. In talking about the dangers from cognitive enhancement for example they say:

… it is enough if very few of us are malevolent or vicious enough to use this power for all of us to run an unacceptable increase of the risk of death and disaster. To eliminate this risk, cognitive enhancement would have to be accompanied by a moral enhancement which extends to all of us, since such moral enhancement could reduce malevolence.^41^

Here they are explicit that cognitive enhancement ‘would have to be accompanied by a *moral* enhancement which extends to *all* of us’ before the risks of cognitive enhancement would be other than ‘unacceptable’.

Notice also that they talk of the *elimination* of the risk in a context where moral enhancement which *extends to all of us* will only, at best, *reduce* malevolence. So even in the most ideal scenario in their terms, a risk which obtains if only one malevolent person escapes is still, for them, massively significant. So we have a strategy to eliminate a danger which has to be universally applied but only reduces (but not eliminates) the risk of the danger, the risk of which risk is defined in terms of the people who pose the danger (whose numbers are already admittedly very small) - ‘it is enough if *very few* of us are malevolent’!

But of course cognitive enhancement is also well calculated to speed up the sorts of advances that can, do and will save lives. We would have to be very sure of the probability of its negative effects to be justified in ignoring the positive ones.

My own reading of the balance here is rather different. Science, innovation and knowledge production, particularly education, are I believe our chief hope of finding solutions to the most threatening sources of probable mass destruction and are moreover our only proven form of moral enhancement to date (and have proved very effective). Add to this the point, emphasized by Martin Rees, that evil is not the most probable source of catastrophe, and that threats not amenable to moral enhancement such as asteroids, new diseases, climate change, and idiocy, inadvertence, and negligence of all sorts, are equally likely, perhaps more likely, to produce disaster, and we have a different agenda. This agenda is to reject the idea of putting cognitive enhancement on hold until moral enhancement is in place to rein it in. Rather we must embrace reliable forms of cognitive enhancement in the hope and reasonable expectation that they are our best prospect of self-defence, including whatever element of self-defence may eventually result from moral enhancement. Indeed cognitive enhancement might reasonably be expected to reduce idiocy, even of the common or village variety!

Milton reminded us that we have been made with both freedom and a powerful sense of justice and the right, and I am sure that Darwinian evolution rather than God is the force responsible. Either way we have what we need, both to know the good and to try to do the good. This knowledge, like all knowledge, can be improved upon and I believe we should look to improve our capacity for knowledge as effectively and as fast as possible. But the other part of Milton's insight is the crucial role of personal liberty and autonomy: that sufficiency to stand is worthless, literally morally bankrupt, without freedom to fall. Again my own view is that I, like so many others, would not wish to sacrifice freedom for survival. I might of course lack the courage to make that choice when and if the time comes. I hope however that I would, and I believe, on grounds that have more eloquently been so often stated by lovers of freedom throughout history, that freedom is certainly as precious, perhaps more precious than life.

Perrson and Savulescu end their paper with a truly chilling reference to C.S. Lewis's stories for children and the frightful ‘Deplorable Word’.

This is a ‘magical curse which ends all life in the world except that of the one who speaks it’ … If we all knew the Deplorable Word, the world would likely not last long. The Deplorable word may arrive soon, in the form of nanotechnology or biotechnology. Perhaps the only solution is to engineer ourselves so that we can never utter it, or never want to utter it.^42^

Ironically and perhaps self-defeatingly it would have to be biotechnology, and possibly cognitively enhanced biotechnology, that would give us the power to engineer ourselves into losing our freedom to innovate in biotechnology in this truly deplorable way. I think we have to hope for something better and perhaps someone better than C.S. Lewis to analyse the dangers.

Perrson and Savulescu may well be right, but we will only know that once moral enhancement has been perfected and only if thereafter bad men, madmen and idiots have ceased to commit or attempt acts of mass destruction. The perfection of moral enhancement is admitted by Perrson and Savulescu to be ‘not likely to be possible in the near future’. I believe it will never be possible to the extent the Perrson/Savulescu thesis requires, or indeed that Tom Douglas believes, both for reasons already given and because moral enhancement has little prospect of preventing idiocy – but of course I could be wrong! Even if Perrson and Savulescu have made the better bet, we will have to wait a long time to know which of us is right, a long time for non-traditional moral enhancement to be possible and then another, possibly even longer, period of time for it to become universal and then another even longer time than that to have any idea whether or not it is working. Meanwhile wickedness and idiocy, not to mention human inadequacy, will continue to grind out their daily death toll, a toll that might in all this time have been dramatically reduced by discoveries accelerated by cognitive enhancement. Moreover, these discoveries might save (might have saved) us from a very large class of mass destruction not attributable to malice, and therefore not susceptible to moral enhancement, such as disease, asteroid strikes and climate change. I don't believe it would be rational to bet on moral enhancement and against accelerating our ability to deal with … literally anything, an ability which is likely to stem, with immediate effect, from cognitive enhancement whether it takes the form of greater alertness or wakefulness in pilots and long distance drivers and emergency medical staff or better decision making from workers who have to function in all sorts of demanding situations.^43^

A strategy that leaves us free to search for solutions to problems we cannot as yet even foresee, one that permits us to use techniques of cognitive enhancement to accelerate that process and one which leaves us free to find, and equipped to implement, those solutions as quickly as possible is a better bet. It is surely better to remain sufficient to stand and to hang on to our precious freedom to fall.

## Footnotes

1 John Milton. 2000. [1667]. *Paradise Lost.* In *Paradise Lost*. J. Leonard, ed. London: Penguin Books.

2 Listen to, yes! Worship – certainly not!

3 See: F. Fukuyama. 2002. *Our Posthuman Future.* London: Profile Books; & J. Habermas. 2003. *The Future of Human Nature.* Cambridge: Polity Press. It is unusual for me to find God on my side, so I am grateful to Milton for listening so intently.

4 John Harris. 2007. *Enhancing Evolution.* Princeton, NJ & Oxford: Princeton University Press.

5 For the significance of hot baths see: Simone Weil. 1966. The Illiad, A Poem of Force. In *The Pacifist Conscience.* Peter Meyer, ed. Harmondsworth: Penguin Books.

6 See for example John Harris, ed. 2001. *Bioethics*. Oxford Readings in Philosophy. Oxford & New York: Oxford University Press. See especially the Introduction.

7 Ibid: line 96ff.

8 William Golding. 1959. *Free Fall.* London; Faber and Faber.

9 While a graduate student in Oxford I had the good fortune to get to know Bill Golding through my friendship with his daughter Judy and with Terrel Carver (later to become Bill's son-in-law) and had the privilege of discussing freedom and many other issues with Golding on a number of occasions.

10 Golding, *op. cit*. note 3, p. 5 of 1966 edn. Harmondsworth: Penguin Books.

11 Not ‘freedom from’ but ‘freedom to’. See Isaiah Berlin. 1969. *Two Concepts of Liberty.* In I. Berlin. *Four Essays on Liberty.* Oxford: Oxford University Press.

12 For example in two essays typical of, indeed exemplary of, recent work on moral enhancement, see below.

13 See John Harris. 1980. *Violence and Responsibility.* London: Routledge & Kegan Paul.

14 In this section on moral enhancement I am influenced by the fact that I have just finished discussing these questions in a new introduction to the paperback edition of my book, Harris, *op. cit.* note 4.

15 Thomas Douglas. Moral Enhancement. *J Appl Philos* 2008; 25(3). See also http://www.ncbi.nlm.nih.gov/pubmed/19132548[Accessed 11 Apr 2010].

16 Ibid: 231.

17 Ingmar Perrson & Julian Savulescu. The Perils of Cognitive Enhancement and the Urgent Imperative to Enhance the Moral Character of Humanity. *J Appl Philos* 2008; 25(3).

18 Ibid: 168.

19 I am grateful to Sarah Chan for reminding me of this important point.

20 Perrson & Savulescu, *op. cit.* note 12, p. 172.

21 Peter Strawson. Freedom and Resentment. *Proc Br Acad* 1960: 48.

22 Douglas, *op. cit*. note 10.

23 I. Perrson & J. Savulescu. The perils of Cognitive Enhancement and the Urgent Imperative to Enhance the Moral Character of Humanity. *J Appl Philos* 2008; 25(3).

24 Elizabeth Fenton has also criticized the Perrson and Savulescu arguments against cognitive enhacement, although she is more sanguine than I am as to the possible merits and success of genetic or biological cognitive enhancement. See Elizabeth Fenton. The Perils of Failing to Enhance: a Response to Perrson and Savulescu. *J Med Ethics* 2010; 36(3): 148–151.

25 Perrson & Savulescu, *op. cit.* note 18, p. 173.

26 http://www.guardian.co.uk/world/2009/dec/27/abdul-muttalab-flight-253-terrorist-al-qaida. [Accessed 11 Apr 2010].

27 http://news.bbc.co.uk/1/hi/uk/8431499.stm[Accessed 11 Apr 2010]

28 http://en.wikipedia.org/wiki/Richard_Reid_(shoe_bomber)[Accessed 11 Apr 2010].

29 http://news.bbc.co.uk/1/hi/health/8606032.stm[Accessed 11 Apr 2010].

30 sites:http://www.infoplease.com/cig/dangerous-diseases-epidemics/smallpox-12000-years-terror.html[Accessed 11 Apr 2010].

31 http://www.who.int/mediacentre/factsheets/smallpox/en/[Accessed 11 Apr 2010].

32 Martin Rees. 2004. *Our Final Century.* London: Arrow Books.

33 Ibid: 61.

34 As reported in 2006 *Guardian Unlimited* 29 May. Available at: http://blogs.guardia.co.uk/culturevulture/archives/2006/05/29/doom_destructio.html[Accessed 8 Apr 2010].

35 Donald Rumsfeld was by no means the first to give this memorable truism an epigrammatic handle. The British Prime Minister, Harold MacMillan, gave this idea slightly more elegant expression in response to a journalist who allegedly asked him what might blow the government off course was ‘events dear boy, events’ and much as it goes against the grain they were both right.

36 John Harris. 1980. *Violence and Responsibility.* London: Routledge & Kegan Paul.

37 W. Barrington Moore Jr. 1969. *The Social Origins of Dictatorship and Democracy*. Harmondsworth: Penguin: 103.

38 Ibid.

39 See for example J. Savulescu. Justice, Fairness and Enhancement. *Ann N Y Acad Sci* 2006; 1093: 321–338.

40 Fenton, *op. cit.* note 19, p. 149.

41 Perrson & Savulescu, *op. cit.* note 18, p. 166.

42 Perrson & Savulescu, *op. cit.* note 18, p. 175.

43 Henry Greely *et al*. Towards Responsible Use of Cognitive Enhancing Drugs by the Healthy. *Nature* 2008; 456: 18–25.

